# Psychometric properties of the Connor-Davidson Resilience Scale (CD-RISC) in a non-clinical population in Sweden

**DOI:** 10.1186/s12955-020-01383-3

**Published:** 2020-05-12

**Authors:** Katarina Velickovic, Ingalill Rahm Hallberg, Ulrika Axelsson, Carl A. K. Borrebaeck, Lisa Rydén, Per Johnsson, Johanna Månsson

**Affiliations:** 1 Department of Psychology, Lund University, Box 213, 22100 Lund, Sweden; 2 Department of Health Sciences, Lund University, Box 157, 22100 Lund, Sweden; 3 Department of Immunotechnology and CREATE Health Translational Cancer Center, Lund University, Medicon Village, 223 81 Lund, Sweden; 4 Department of Clinical Sciences Lund, Division of Surgery, Lund University, Medicon Village, 223 81 Lund, Sweden; 5 Department of Surgery, Skåne University Hospital, Lund, Södra Förstadsgatan 1, 214 28 Malmö, Sweden

**Keywords:** Psychological resilience, Emotion regulation, Health-related quality of life, CD-RISC, DERS-16, SF-12, Construct validity, Discriminant validity, Predictive validity

## Abstract

**Background:**

The Connor-Davidson Resilience Scale (CD-RISC) is the most widely used scale which assesses psychological resilience. Although it is recommended to be applied as a unidimensional scale, its factor structure, reliability, as well as discriminant and predictive validity need to be assessed when used in a new context. Moreover, the original five-factor structure has not been replicated in previous investigations. This study aimed to explore psychometric properties of the scale in a Swedish context.

**Methods:**

Construct validity of the five-factor model of CD-RISC was assessed using Exploratory and Confirmatory Factor Analyses. Its discriminant validity was assessed in relation to a measure of emotion regulation (Brief Version of the Difficulties in Emotion Regulation Scale) using a Confirmatory Factor Analysis. Predictive validity of CD-RISC was assessed in relation to measures of physical and mental health-related quality of life (The 12-Item Short Form Survey) using hierarchical multiple regression analyses. A population based sample cohort was employed (*N* = 2599).

**Results:**

Exploratory and Confirmatory Factor Analyses suggested a 22-item unidimensional model of CD-RISC. Psychological resilience was found to be independent from the measure of emotion regulation. It was shown to predict both physical and mental health-related quality of life, being especially strongly associated with mental health aspects.

**Conclusions:**

The study showed that the Swedish version of CD-RISC is an instrument with high discriminant and predictive validity, although the original factor structure does not apply in this context. CD-RISC can thus be used to identify individuals with a higher need of psychosocial support, especially relating to mental health needs.

## Background

Even though many definitions of psychological resilience (further referred to as resilience) have been suggested, there is a general consensus that it implies the presence of *positive adaptation* in face of *significant adversity* [1]. The importance of resilience for overall wellbeing, health, and a multitude of other positive outcomes, has been demonstrated within many areas [2–4]. The most widely used scale of resilience, the Connor-Davidson Resilience Scale (CD-RISC) [5] has, to the best of our knowledge, not been validated in a Swedish context. Given the utility of resilience in predicting a multitude of positive outcomes, as well as the unique characteristics of the Swedish context, it is crucial to explore its psychometric properties prior to applying it in research and clinical settings.

Resilience is a complex concept and there has been a great variability in the ways it has been operationalized and studied over the years. First investigated in the context of developmental psychology [6, 7], resilience has subsequently been explored in a variety of contexts, which vary from mild hassles (e.g., work stress) to severe trauma (e.g., bereavement, life threatening events) [8]. Many factors contribute to resilience, including personal factors, such as as spirituality, optimism, positive emotions, cognitive flexibility, active coping, and acceptance [9], as well as biological [10] and environmental factors (e.g., relationship with family and peers, social belonging) [11], which can be considered as sources of resilience. Many scales for measuring resilience have been developed. Some of the existing measures include The Dispositional Resilience Scale [12], The Resiliency Attitudes and Skills Profile [13], The Resilience Scale [14], Psychological Resilience [15], the Resilience Scale for Adults [16], The Brief Resilience Scale [17], and the Connor-Davidson Resilience Scale [5]. In a review by Windle, Bennett, and Noyes, it was found that the last three scales mentioned above have the best psychometric properties (i.e., content validity, internal consistency, criterion validity, construct validity, reproducibility, agreement, reliability, responsiveness, floor and ceiling effects, and interpretability). However, none of the scales seem to have higher than moderate psychometric quality [18].

The scale which has been particularly widely used is CD-RISC. CD-RISC contains 25 items, which are rated on a five-point Likert scale and range from 0 (“Not true at all”) to 4 (“True nearly all the time”). Possible scores thus range from 0 to 100. Connor and Davidson found that these items correspond to five factors [5]. The first factor reflects having high standards, tenacity, and competence (eight items). The second factor reflects handling negative emotions, trusting one’s instincts, and perceived benefits of stress (seven items). The third factor reflects having a positive attitude to change and secure relationships (five items). The fourth one reflects perceived control (three items), and the fifth one spirituality (two items).

Nevertheless, when applied in a new context, it is necessary to explore construct, discriminant, and predictive validity of CD-RISC. For example, the five-factor structure found in the original study [5] has not been replicated in subsequent explorations. Whereas some studies revealed two [19], three [20, 21], or four [22, 23] factor models, most identified studies unveiled a unidimensional structure of the scale, sometimes retaining only 22 [24, 25] or 10 [26, 27] items. Moreover, several issues in relation to the scale have been identified, such as the first three factors being thematically heterogeneous, and the fourth and fifth factor containing only three and two items respectively.

It can be argued that the structure of CD-RISC is at least partially dependent on the context and population in which it is administered. There have yet been no validation studies of CD-RISC in Sweden. It could be suspected that sources of resilience in Sweden would differ as compared to other populations. For instance, the percentage of population belonging to the Church of Sweden has been decreasing every year, dropping down to 57.7% in 2018 [28]. Additionally, according to a report from 2012, only 29% of surveyed Swedish citizens claimed they held religious beliefs [29]. Therefore, it could be expected that the “Spirituality” factor, which has previously been discussed as problematic, especially item 3 (“Sometimes only God can help”), would not constitute an important source of resilience in this population. Thus, the question remains whether the construct resilience can be operationalised, in a Swedish context, using the present CD-RISC structure, and if it performs well in relation to proposed related measures.

### Resilience and emotion regulation

Aiming to assess psychometric properties of CD-RISC, it is important to understand how this instrument operates in relation to related, but distinct concepts. One of those concepts is emotion regulation, which refers to the processes by which we shape which emotions we have, when we have them, how we experience them and how we express them [30]. People regulate both positive and negative emotions by employing a variety of strategies. As previously noted, adversity, one of the key ingredients of the theoretical construct of resilience, involves stressful and/or life-threatening situations, which can instigate strong negative emotions. It is thus plausible that resilient people, who manage to maintain normal levels of functioning when confronted with an emotion-laden situation, also regulate their emotions more effectively.

In line with that, previous research suggested that using more adaptive emotion regulation strategies is one of the protective factors which contribute to resilience [31]. Cognitive reappraisal, one of adaptive emotion regulation strategies, has been shown to predict positive and negative outcomes in people dealing with stressful situations. For example, using this strategy had a buffering effect of stress on negative outcomes in caregivers for people with multiple sclerosis [32], and predicted positive emotions among people caring for individuals with AIDS [33]. Furthermore, cultivating positive emotions has been suggested as one of the mechanisms resilient people use to bounce back from stressful events [34, 35]. It was thus proposed that using adaptive emotion regulation strategies can moderate the relationship between a stressor and resilient outcomes [31].

As noted earlier, however, resilience has been operationalised as a complex construct which comprises not only successful emotion regulation, but a variety of other protective factors and cognitive mechanisms, such as social relationships, coping, cognitive flexibility, and acceptance [9]. Therefore, emotion regulation and resilience should represent closely related, but nevertheless different concepts. In this study, discriminant validity of CD-RISC was thus explored by examining its independence from emotion regulation.

### Resilience and health-related quality of life

To assess predictive validity of CD-RISC, it is crucial to explore its relationship with a concept expected to be predicted by resilience, such as health-related quality of life (HRQoL). As the core ingredient of resilience is positive adaptation, it is reasonable to assume highly resilient people experience more positive outcomes and have a higher HRQoL than those who are low on resilience when confronted with a stressful life situation. One of the definitions of HRQoL suggests that it constitutes one’s perceived wellbeing relating to mental, physical, and social aspects of health [36]. Resilience has been associated with various health outcomes in both healthy populations and populations with various health conditions. For example, it has been shown to predict physical health in patients with diabetes [37], HRQoL in patients with liver disease [38], secondary symptoms in people with long-term disability [39], physical activity levels in cancer patients [40], physical health in survivors of hematopoietic cell transplantation [41], but also chronic pain and physical health among older people [42]. Moreover, resilience has consistently been associated with mental health outcomes [43] among cancer patients [40], survivors of hematopoietic cell transplantation [41], older people [42], young adults [44], and athletes [45]. Therefore, physical and mental HRQoL were included in the present study as an outcome of resilience, with the aim to test predictive validity of CD-RISC.

### Aim

The aim of the present investigation was to determine construct validity of the five-factor model of CD-RISC, its internal consistency, as well as discriminant and predictive validity in relation to measures of emotion regulation and HRQoL respectively, in a Swedish context.

## Method

### Procedure

Data collection was obtained within the BIG3 project [46], which aims to explore various health aspects of inhabitants of the region of Skåne in Sweden, focusing on pulmonary disease, cardiovascular diseases, and lung cancer. Within this longitudinal project, data collection was performed in three rounds. First, 57,107 randomly selected people in Skåne, aged 45–75, received a screening questionnaire by post and were invited to participate in the study. Out of those, 11,083 agreed to take part. From this sample, 5230 participants took part in the second round of the study, which included examinations and a more extensive questionnaire. This subsample was selected randomly, except that it was geared towards achieving a distribution of 25% smokers, 25% non-smokers, and 50% former smokers. Finally, 3724 randomly selected participants that had concluded the second round were invited to respond to a follow-up questionnaire, which was conducted online and was when the measures included in this study were obtained. At the time when the data was obtained for the current study, 2604 participants had responded (i.e., the retention rate was 69.9%).

### Measures

#### Resilience

The content, structure, and properties of CD-RISC have been described in the previous section. In this study, a Swedish version of the scale has been used. Higher scores on CD-RISC indicate higher levels of resilience.

#### Emotion Regulation

Brief Version of the Difficulties in Emotion Regulation Scale (DERS-16) [47] contains 16 items, which are rated on a 5-point Likert scale and range from 1 (“Almost never”) to 5 (“Almost always”). It measures difficulties in different aspects of emotion regulation, such as engaging in goal-directed behaviour (e.g., “When I’m upset, I have difficulty focusing on other things”), accessing various emotion regulation strategies (e.g., “When I’m upset, I believe that I’ll end up feeling very depressed”), and impulse control (e.g., “When I’m upset, I feel out of control“). Possible scores range from 16 to 80, higher scores indicating higher emotion dysregulation. A Swedish version of DERS-16 was employed and had high internal consistency (α = .92).

#### Health Related Quality of Life

The 12-Item Short Form Survey [48] measures one’s perception of different health aspects. It comprises 12 items and provides two summary component scores – the Physical Component Summary Score (PCS12) and the Mental Health Component Summary Score (MCS12). In this study, a Swedish version of the scale previously used in the BIG3 study was employed.

#### Demographic variables

Participants also stated their age, gender, as well as highest level of education. Socioeconomic status (SES) was measured by asking how often participants have difficulties paying the bills.

#### Health variables

Participants also reported whether they were diagnosed with some of 18 predefined health conditions.^1^ Additionally, their smoking habits were recorded.

### Data analysis

The sample was randomly divided into three subsamples. Exploratory Factor Analysis (EFA) was performed in order to see which factors will emerge in this context. It was performed on the two subsamples and the results were compared to explore the stability in factor structure. Principal Axis Factoring (PAF) and promax rotation were employed, as oblique rotations are recommended when factors are expected to be moderately or highly correlated [49]. EFA were performed on the polychoric correlations matrices, as data are Likert-based. Additionally, Parallel Analysis was performed to guide the EFA extraction. Parallel Analysis compares the obtained eigenvalues to those acquired by randomly generating 1000 polychoric correlation matrices [50]. Subsequently, the acquired model was tested on the third subsample with a Confirmatory Factor Analysis (CFA). After that, discriminant validity of CD-RISC in relation to DERS-16 was performed utilising a CFA on the third subsample. A hypothesised two-factor structure was thereby compared to a one-factor structure. The standard Goodness-of-Fit Indices criteria were utilised when assessing final models (RMSEA *<*.08; SRMR *<*.05; CFI *>* .90), and AIC scores for both models were compared. In both CFAs, instead of factor loadings of first indicators, variances of latent variables were fixed to one. The Sattora-Bentler scaled test statistic was used as a correction, as the maximum-likelihood (ML) method for parameter estimation requires a normal distribution. Therefore, robust estimators and indices were reported.

Finally, predictive validity was assessed on the total sample with two hierarchical multiple regression analyses, aiming to test whether resilience predicts physical health above and beyond sociodemographic and health variables. In both hierarchical multiple regression analyses, significant sociodemographic and health variables were included in the first step of the analyses, whereas CD-RISC was added in the second step. PCS12 and MCS12 were included as dependent variables. Scores on PCS12 and MCS12 were calculated using a syntax containing regression weights recommended for the Swedish population. As one of the items in the questionnaire (item 12) contained six response categories instead of five, as intended in the original questionnaire, the redundant response category (i.e., category three) was recoded into 2.5, and multiplied by regression weights for categories 2 and 3. The obtained score distribution for this item was thereby not significantly altered. All analyses were performed using the RStudio Software, version 1.1.456 [51].

## Results

### Sample characteristics

The initial sample included 2604 participants from a contemporary population from rural and non-rural settings, residing in the region of Skåne in Sweden. Five participants were, however, excluded from the analysis, as they only responded to 13% of the survey items. Thus, a total of 2599 participants were retained in the final sample. Additionally, there were 209 (8.04%) missing data on the SES variable. Participants who did not answer this question had lower scores on PCS12 (t(228.99) = 10.27, *p* < .001, d = 0.82), MCS12 (t(237.76) = 2.02, *p* < .05, d = 0.15), as well as CD-RISC (t(236.55) = 2.49, *p* < .05, d = 0.19). Sample characteristics are provided in Table 1.

**Table 1:** Sample characteristics (*N* = 2599)

Characteristic	*N*	%
Gender		
Male	1316	50.63
Female	1283	49.36
Age		
45–54	330	12.70
55–64	737	28.36
65–74	1283	49.36
75–84	249	9.58
Education level completed		
Unfinished primary	6	0.23
Primary	393	15.12
Secondary	1126	43.32
Tertiary	1070	41.17
Health Condition		
Yes	1018	39.17
No	1581	60.83
Smoking		
Quit smoking	1262	48.56
Never smoked	922	35.47
Sometimes smokes	95	3.65
Smokes daily	319	12.27
Difficulties paying the bills (SES)		
Never	2223	85.53
Sometimes	124	4.78
About half of the months	19	0.73
Every month	24	0.92

The sample was split into three subsamples (*n1* = 866, *n2* = 866, *n3* = 867). Means and Standard Deviations on four main measures (i.e., CD-RISC, DERS-16, PCS12, and MCS12) across the subsamples are provided in Table 2.

**Table 2:** Means (M) and standard deviations (SD) for subsample 1 (*n* = 866), subsample 2 (*n* = 866), and subsample 3 (*n* = 867)

Measure	Subsample 1		Subsample 2		Subsample 3	
	M	SD	M	SD	M	SD
1. CD-RISC	68.71	13.24	68.88	12.61	68.88	13.07
2. DERS-16	25.80	9.03	25.42	8.75	25.52	8.45
3. PCS12	48.02	9.46	48.34	9.44	48.65	9.02
4. MCS12	52.89	9.81	53.48	8.91	53.56	9.03

As presented in Table 3, the distribution of CD-RISC was negatively skewed, with the average scores being higher than the theoretical mean (i.e., 50). There were no differences in resilience scores among men and women. The distribution of DERS-16 was positively skewed, with the majority of participants scoring low on this scale. Moreover, there was a significant difference across genders, with women scoring higher than men (t(2597) = 5.27, *p* < .001, d = 0.21), but the effect size was small [52]. Distributions of PCS12 and MCS12 were negatively skewed. Gender differences were significant on both PCS12 (t(2597) = 3.58, *p* < .001, d = 0.23) and MCS12 (t(2597) = 3.66, *p* < .001, d = 0.14), but effect sizes were small [52].

**Table 3:** Means (M), standard deviations (SD), standardised skewness, and standardised kurtosis for variables (*N* = 2599)

Measure	Male		Female		Standardised skewness	Standardised kurtosis
	M	SD	M	SD		
1. CD-RISC	68.92	12.37	68.72	13.55	- 4.94***	1.97*
2. DERS-16	24.69	8.11	26.49	9.27	40.50***	56.06***
3. PCS12	49.73	7.90	47.67	9.88	−48.65***	9.02***
4. MCS12	53.92	9.04	52.64	9.65	53.56***	9.03***

*p < .05. ***p < .001

Finally, out of 18 health conditions which were measured in the study, only four had a significant, but small effect on CD-RISC scores – hypertension (*t*(2597) = 3.13, *p* < .01, *d* = .13), high cholesterol (*t*(2597) = 2.68, *p* < .01, *d* = .13), stroke, blood clots in the brain, or cerebral haemorrhage (*t*(2597) = 2.11, *p* < .05, *d* = .28), and chronic obstructive pulmonary disease (*t*(2597) = 4.11, *p* < .001, *d* = .43).

### Construct validity of CD-RISC

Parallel analysis suggested five factors should be extracted. Therefore, EFAs were performed on subsamples 1 and 2 and five factors were initially extracted. However, a few problems emerged. In both subsamples, two of the obtained factors contained only three (i.e., items 3, 9, and 20) and two items (i.e., items 2, 13) respectively. Also factor stability across two subsamples was low, as these were the only factors which had the same factor loadings across two subsamples (the criterion loading of ≤ .32 was used). In addition, correlations between three factors were moderate to high (r > 0.6). Finally, two items cross-loaded on different factors (i.e., items 10 and 11 on subsample 1; items 11, 12, 14, and 15 on subsample 2).

Consequently, we specified a four-factor solution. However, similar problems emerged. Most of the items loaded on factor 1 and 2, and on subsample 2 item 10 did not load on any factors. Factor stability was again low and three factors had moderate to high correlations (r > 0.6). To address these problems, a three-factor model was extracted, but it resulted in the same issues. Therefore, the exploration proceeded with a two-factor model. This model showed good factor stability across two subsamples. However, factor 2 contained only three items (i.e., 3, 9, and 20), whereas other items loaded on factor 1. Finally, a one-factor model was tested and resulted in items 3 and 9 not loading on the extracted factor on neither subsamples, whereas item 20 loaded on the extracted factor on the subsample 1, but not the subsample 2 (Table 4). These items were thus excluded and the unidimensional model was rerun on the remaining 22 items. This model showed good factor stability, as all items had loadings > .32 on both subsamples. The model explained 47 and 45% of the variance on subsamples 1 and 2 respectively.

**Table 4:** Factor loadings on subsamples 1 (*n* = 866) and 2 (*n* = 866) CD-RISC items using a one-factor solution

Item	Factor loadings	
	Subsample 1	Subsample 2
1. Able to adapt to change	0.65	0.53
2. Close and secure relationships	0.35	0.35
**3. Sometimes fate and God can help**	**0.08**	**0.09**
4. Can deal with whatever comes	0.72	0.68
5. Past success gives confidence for new challenges	0.80	0.80
6. See the humorous side of things	0.61	0.58
7. Coping with stress strengthens	0.64	0.64
8. Tend to bounce back after illness or hardship	0.71	0.70
**9. Things happen for a reason**	**0.25**	**0.23**
10. Best effort no matter what	0.57	0.57
11. You can achieve your goals	0.81	0.78
12. When things look hopeless, I don’t give up	0.76	0.70
13. Know where to turn for help	0.58	0.61
14. Under pressure, focus and think clearly	0.71	0.70
15. Prefer to take the lead in problem solving	0.66	0.64
16. Not easily discouraged by failure	0.57	0.57
17. Think of self as strong person	0.82	0.79
18. Make unpopular or difficult decisions	0.62	0.60
19. Can handle unpleasant feelings	0.72	0.70
**20. Have to act on a hunch**	**0.38**	**0.32**
21. Strong sense of purpose	0.75	0.78
22. In control of your life	0.71	0.72
23. I like challenges	0.74	0.76
24. You work to attain your goals	0.73	0.77
25. Pride in your achievements	0.67	0.67

Items with a factor loading ≤ .32 are in bold

With the aim to test the acquired 22-item unidimensional model, a CFA was performed on subsample 3 (*n* = 867). Robust estimators and indices are presented in the table below. Despite the *χ2* being significant (*χ2* (2, *n* = 867) = 955.58, *p* < .001), both absolute and relative fit indices suggested that a one-factor model fits the data well (RMSEA = 0.06, SRMR = 0.05, CFI = 0.89). Moreover, Table 5 indicates that all items had loadings > .32 on the proposed factor (item 2 having a borderline .32 loading). The results of the CFA thus support the model obtained in the EFAs. Internal consistency of the 22-item CD-RISC was high (α = .91).

**Table 5:** Unstandardised and standardised loadings for the one-factor model for CD-RISC items (*n* = 867)

Item	Unstand.	SE	Stand.
1. Able to adapt to change	0.37	0.02	14.75
2. Close and secure relationships	0.32	0.05	6.9
4. Can deal with whatever comes	0.48	0.02	19.32
5. Past success gives confidence for new challenges	0.58	0.02	26.04
6. See the humorous side of things	0.47	0.03	16.40
7. Coping with stress strengthens	0.57	0.03	17.15
8. Tend to bounce back after illness or hardship	0.38	0.02	18.57
10. Best effort no matter what	0.33	0.02	18.09
11. You can achieve your goals	0.55	0.02	25.41
12. When things look hopeless, I don’t give up	0.52	0.02	23.91
13. Know where to turn for help	0.51	0.03	16.02
14. Under pressure, focus and think clearly	0.66	0.03	23.56
15. Prefer to take the lead in problem solving	0.57	0.03	20.03
16. Not easily discouraged by failure	0.46	0.03	13.52
17. Think of self as strong person	0.65	0.03	25.33
18. Make unpopular or difficult decisions	0.63	0.03	21.06
19. Can handle unpleasant feelings	0.56	0.03	21.18
21. Strong sense of purpose	0.63	0.03	23.38
22. In control of your life	0.55	0.03	20.52
23. I like challenges	0.68	0.03	26.54
24. You work to attain your goals	0.65	0.02	27.29
25. Pride in your achievements	0.54	0.02	22.05

### Discriminant validity of the 22-item CD-RISC

Further, discriminant validity of CD-RISC was investigated by exploring its relationship with DERS-16. A CFA was performed on the subsample 3 (*n* = 867), utilising 22 items which were retained in the final model, as well as 16 items comprised within DERS-16. A two-factor structure was compared to a one-factor structure. Due to the issues with normality of the data, robust estimators and indices are reported.

Despite obtaining significant *χ2,* CFA revealed that the two-factor model was a better fit with the data on all Goodness-of-Fit Indices (Table 6). Moreover, all items had a loading >.32. This finding supports the notion that, despite being correlated, resilience and emotion regulation represent two separate constructs.

**Table 6:** Goodness-of-Fit indices for one- and two-factor solutions for CD-RISC and DERS-16 items (*n* = 867)

Model	*χ2*	*df*	RMSEA	SRMR	CFI	AIC
One-factor	4287.42***	665	0.08***	0.10	0.61	69,798.19
Two-factor	2239.25***	664	0.05*	0.05	0.83	66,746.72

*p < .05. ***p < .001

### Predictive validity of CD-RISC

#### Physical health-related quality of life

Hierarchical multiple regression was performed to test the predictive utility of CD-RISC above significant sociodemographic and health variables. Gender, age, education, smoking, SES, and presence of one of four health conditions which had a significant effect on resilience were added in the first step of the regression analysis, whereas the 22-item CD-RISC score was added in the second step. Overall, specified multiple regression model explained 7.4% of the variance (F(7,2378) = 28.27, *p* < .001, Adj. R^2^ = 0.07). Moreover, addition of CD-RISC significantly improved prediction (F change (12378) = 71.34, *p* < .001, *R*^*2*^ change = 0.03), thus providing support for the hypothesis. All variables except for smoking were significant predictors of physical HRQoL, CD-RISC being the most important predictor (Table 7).

**Table 7:** Summary of the regression model of physical HRQoL (*N* = 2381)

	b	SE	Β	*t*	*p*	2.5%CI	97.5%CI
b0	40.74	2.25	0.00	18.08	.00***	36.32	45.16
Gender	−0.10	0.35	−0.05	−2.89	.00**	−1.68	−0.32
Age	−0.08	0.02	−0.07	−3.61	.00***	−0.13	−0.04
Education	0.56	0.25	0.05	2.21	.02*	0.06	1.05
Smoking	0.34	0.19	0.03	1.78	.10	−0.03	0.71
SES	1.10	0.43	0.09	4.63	.00***	1.15	2.85
Health	−2.11	0.37	−0.15	−5.72	.00***	−2.83	−1.39
CD-RISC	0.12	0.01	0.17	8.45	.00***	0.09	0.15

*p < .05. ***p < .001

#### Mental health-related quality of life

A second hierarchical multiple regression was performed using mental HRQoL as a dependent variable. Gender, age, smoking, and SES were added in the first step of the regression analysis,^2^ and the 22-item CD-RISC score was added in the second step. The model explained 24.69% of the variance (F(5,2383) = 157.56, *p* < .001, Adj. R^2^ = 0.25), all included variables being significant predictors of mental HRQoL (Table 8). Addition of CD-RISC significantly improved prediction (F change (12383) = 567.80, *p* < .001, *R*^*2*^ change = 0.18) and CD-RISC was the strongest predictor.

**Table 8:** Summary of the regression model of mental HRQoL (*N* = 2384)

	b	SE	β	*t*	*p*	2.5%CI	97.5%CI
b0	10.14	2.07	0.00	4.90	.00***	6.08	14.20
Gender	−0.77	0.33	−0.04	−2.36	.01*	−1.41	−0.13
Age	0.22	0.02	0.19	10.48	.00***	0.18	0.26
Smoking	0.68	0.18	0.07	3.86	.00***	0.33	1.02
SES	2.44	0.41	0.11	6.01	.00***	1.64	3.24
CD-RISC	0.33	0.01	0.42	23.83	.00***	0.30	0.35

*p < .05. ***p < .001

## Discussion

The main aim of the current investigation was to assess construct, discriminant, and predictive validity of CD-RISC in a contemporary sample of randomly invited inhabitants of the region of Skåne in Sweden. Overall, it was revealed that CD-RISC was a sound psychometric tool for measuring resilience, with good predictive and discriminant validity. This study also demonstrated that CD-RISC has high internal consistency, and can therefore be used in the clinical context. Moreover, the data showed it to be a unidimensional, rather than a multidimensional instrument, as the original five-factor structure was not replicated in this Swedish sample. On the contrary, EFAs suggested that a 22-item unidimensional model should be retained. Further, CFA showed this model fit the data well. This result is in line with some of the previous explorations of psychometric properties of CD-RISC. Although some studies revealed two or more factors [19–21, 23] most resulted in unidimensional models of resilience [24–27]. It should be noted that in the original study, authors used an orthogonal rotation [5], whereas in the current investigation an oblique rotation method was employed. Even though this may have contributed to differing results, an oblique rotation is recommended when factors are thought to correlate [49], which is why this approach was assessed as more appropriate. Taken together, previous studies along with the current one provide strong evidence that the original five-factor structure of CD-RISC does not replicate in different contexts, and it further supports the notion that only a total score, as opposed to five sub-scores of CD-RISC should be calculated, as suggested by the constructors [5].

The retained model excluded three items, two of which constitute the “Spirituality” factor from the original model. This is in line with some previous investigations, conducted in Australia and Spain, which excluded the same items [24, 25]. Moreover, exclusion of the spirituality-related items confirms the assumption that this factor may not contribute to resilience in a Swedish population, which may be explained by the notion that the population in Sweden is seemingly not as religious as in many other countries. Most notably the item which relates to religious beliefs (“Sometimes fate and God can help”) had the lowest factor loading. Furthermore, the second item witch constitutes the “Spirituality” factor (“Things happen for a reason”) had a similarly low factor loading, whereas the item which pertains to trusting one’s instincts (“Sometimes you have to act on a hunch”) also did not perform well in this sample, albeit having a borderline factor loading (0.38 in sample 1 and 0.32 in sample 2). Additionally, the item which signifies the importance of social support in handling stressful events (“Having close and secure relationships”) had a borderline factor loading of .32 in the CFA, signifying it should be taken with caution in further investigations.

The study further suggested CD-RISC has a high discriminant validity in the given population. Albeit interrelated, emotion regulation and resilience represent distinct concepts [31]. CFA showed CD-RISC measures a different concept from a measure of emotion regulation, i.e. DERS-16. Moreover, CD-RISC has high predictive validity, considering that hierarchical multiple regression analyses suggested it predicts physical and mental HRQoL, above and beyond demographic and health variables. It was especially important for mental health, which is in line with previous studies which showed resilience is consistently positively associated with indicators of mental health [43]. Thus, resilience may serve as a buffer against low HRQoL in various health conditions and could be a useful tool for understanding the effects of difficult health conditions as well as their treatment procedures.

This study has some limitations pertaining to the sample employed. The data were collected within a wider project, which aims at improving treatment and care of people with chronic obstructive pulmonary disease, cardiovascular diseases, and lung cancer. Given the overarching theme of the project, and that the assessment included a variety of health-related questions and measures, it could be assumed that people with poorer health would be more inclined to agree to participate, thus resulting in a lower score on resilience as compared to the general Swedish population. Nevertheless, the analyses showed only four out of 18 conditions had significant effects on CD-RISC scores, and these were small. It is important to note that the health conditions were self-reported by participants, and there may be variations in functional ability within the same health conditions, which could result in differences in resilience. Moreover, although the recruitment of participants was geared towards obtaining a sample of 25% of smokers, smoking had no effect on CD-RISC (*p* > .05). Also, the mean score on CD-RISC obtained in the original study in a non-clinical sample was 80.4 (*SD* = 12.8) [5]. As the difference between the mean score obtained in this study and the one acquired in the original study is quite significant, the question remains if the scores obtained in our study underestimate the resilience scores among a general population in Sweden.

Additionally, it is important to note that the study population came from one region in Sweden, albeit there is no reason to assume this would have a significant influence on overall scores, especially as the education distribution matched that of Sweden in general. Finally, the age targeted in the overall project was between 45 and 75 years, the average age in this sample being 65.62, which is significantly higher than the mean for Sweden (*M* = 41.2) [53]. Although our analysis revealed that age was not associated with CD-RISC scores (*p* > .05), one limitation of our study could be that the results may be applicable only to those aged between 45 and 75 years.

Despite the abovementioned limitations, this study has significant strengths. Notably, it was conducted on a large sample, which allowed to split the overall sample into three subsamples, and perform two EFAs and a CFA. This strengthened the evidence for the proposed unidimensional 22-item model of CD-RISC. Moreover, this was the first time CD-RISC and its relationships with health-related constructs with an established importance for the clinical population were explored in Sweden on such a large sample. Additionally, the study was conducted within a larger health-related project, which enabled to explore the relationship between CD-RISC and 18 health conditions, thus determining those populations for which the scale is most useful. Therefore, the results of this study have the potential to inform both research and applied contexts in Sweden.

## Conclusions

In spite of abovementioned limitations, the current exploration suggests that CD-RISC is a valid unidimensional instrument. It showed high discriminant validity in relation to a measure of emotion regulation, as well as high predictive validity in relation to a measure of physical and mental HRQoL. Considering the evidence which associates resilience with a plethora of positive outcomes, having a solid tool for measuring this complex construct is of great importance. This study has shown that the 22-item version of CD-RISC can be used in clinical and health-related contexts in Sweden. More specifically, this scale can be employed for identifying people who are in greater need of psychosocial interventions due to lower resilience.

## Data Availability

The datasets used and/or analysed during the current study are available from the corresponding author on reasonable request.

## Abbreviations

AIC: Akaike Information Criterion
CD-RISC: Connor-Davidson Resilience Scale
CFA: Confirmatory Factor Analysis
CFI: Confirmatory Factor Index
DERS-16: Difficulties in Emotion Regulation Scale
EFA: Exploratory Factor Analysis
HRQoL: Health-related Quality of Life
MCS12: Mental Health Component Summary Score
PAF: Principal Axis Factoring
PCS12: Physical Component Summary Score
RMSEA: Root Mean Square Residual
SF-12: The 12-Item Short Form Survey
SRMR: Standardized Root Mean Squared Residual

## Footnotes

1 Health conditions included: Asthma, hypertension, high cholesterol, heart attack, angina, atrial fibrillation, heart failure, stroke, chronic obstructive pulmonary disease, tuberculosis, other lung disease, diabetes, gluten intolerance/celiac disease, inflammatory bowel disease, eczema, psoriasis, cancer, and rheumatic diseases.

2 Education and presence of a health condition were not associated with mental HRQoL (*p* > .05).
